# Comparison of epidural tramadol-ropivacaine and fentanyl-ropivacaine for labor analgesia: A prospective randomized study

**DOI:** 10.3109/03009734.2011.601532

**Published:** 2011-10-29

**Authors:** Yunxia Fan, Muhuo Ji, Lina Zang, Wenhui Wang, Qi Yin, Jian Xu, Jianjun Yang

**Affiliations:** ^1^Department of Anaesthesiology, Jintan Hospital, Jiangsu University, Changzhou, P. R. China; ^2^Department of Anaesthesiology, Jinling Hospital, School of Medicine, Nanjing University, Nanjing, P. R. China

**Keywords:** Analgesia, epidural, fentanyl, labor, tramadol

## Abstract

**Background:**

To test the hypothesis that 5 mg/mL tramadol is superior to 3 μg/mL fentanyl when combined with 0.125% ropivacaine in parturients undergoing labor during epidural analgesia.

**Methods:**

Sixty-one parturients undergoing labor selected for delivery with epidural analgesia were randomized into two groups: Group tramadol (0.125% ropivacaine plus tramadol 5 mg/mL) and Group fentanyl (0.125% ropivacaine plus fentanyl 3 μg/mL). Hemodynamics, rate of cesarean delivery, sensory block level, Bromage motor scale scores, instrument-assisted delivery, oxytocin use, visual analog scale (VAS) scores, Apgar scores, umbilical cord artery gas analysis, and maternal side-effects including nausea, vomiting, pruritus, urinary retention, shivering, hypotension, and respiratory depression were recorded.

**Results:**

The two groups had no significant differences with respect to maternal hemodynamics, neonatal heart rate, VAS scores, rate of cesarean delivery, sensory block level, Bromage motor scale scores, instrument-assisted delivery, oxytocin use, hypotension, nausea, vomiting, and respiratory depression (*p* > 0.05). The incidence of pruritus, shivering, and urinary retention were more commonly observed in Group fentanyl despite there was no significant difference between the two groups. Umbilical artery pH was significantly lower while PCO_2_ was higher in Group fentanyl than Group tramadol (*p* = 0.003 and *p* = 0.026, respectively). Birth-weight, umbilical artery PO_2_ and base deficit, and Apgar scores at 1 and 5 min were comparable between the two groups (*p* > 0.05).

**Conclusions:**

Our observations suggest that tramadol seems to be a safe alternative to fentanyl for labor analgesia due to its similar analgesic efficacy.

## Introduction

Epidural nerve block is widely used for labor analgesia because of its effective pain relief, reduced maternal stress response, improved parturient satisfaction, and potential ability to provide anesthesia (1). The quality of analgesiais improved with the combined use of a local anesthetic and an opioid when combined with the use of either agent alone. An example of acombination epidural therapy that provides excellent sensory block with relatively little motor block includes a co-administration of ropivacaine and fentanyl (2). However, the side-effects of fentanyl are still of concern during its use in perioperative period (3).

Tramadol not only binds to opioid μ-receptors but also interacts with the central nervous system by inhibiting the withdrawal of noradrenaline and serotonin (4). The unique pharmacological profile of tramadol makes it an attractive drug for postoperative pain management. In several preliminary clinical trials, tramadol has been proved to be a safe and effective drug for epidural analgesia (5,6).

Hence, the purpose of the present study was to compare the analgesic and side-effects of two solutions that combined 0.125% ropivacaine with either tramadol 5 mg/mL or fentanyl 3 μg/mL in parturients undergoing labor during epidural analgesia.

## Materials and methods

The Human Investigations Committee of Jintan Hospital approved the present study protocol, and prior written informed consents were obtained from all parturients. A total of 66 nulliparous parturients, with ASA class I or II, eligible for labor epidural analgesia were sequentially enrolled from May 2008 to June 2009 in this prospective, randomized, double-blinded study. Parturients with multiple pregnancies, a history of previous local anesthetic events, hypertension in pregnancy with proteinuria, cardiac diseases, any other major medical disorder associated with pregnancy , and contraindications for epidural analgesia were excluded.

Based on a computer-generated grouping number sheet, the patients were randomized into two groups: Group tramadol (*n* = 33) and Group fentanyl (*n* = 33). The mixed solutions for epidural analgesia were prepared under sterile conditions by an anesthetist and administered by a second anesthetist who remained blinded to the solution prescription. Each parturient received 250 mL lactated Ringer's solution over 20–30 min before epidural injection and was kept in the left lateral position for epidural puncture when cervical dilation was ≥2 cm. The epidural puncture was performed at the L_2–3_ interspace with a mid-line approach and 3 cm of the catheter was placed cephalicly to the left in the epidural space. Aspiration of the in-place catheter for blood or cerebrospinal fluid was done to rule out incorrect placement. Five minutes later, if sensory block level was obtained, a loading dose was added. Group tramadol received 10 mL of 0.125% ropivacaine (AstraZeneca, Södertälje, Sweden) plus 5 mg/mL tramadol (Grunenthal GmbH, Germany),and Group fentanyl received 10 mL of 0.125% ropivacaine plus 3 μg/mL fentanyl (50 μg/mL; Renfu Co., Hubei, China). The aforementioned two solutions were also used for subsequent analgesia maintenance at a constant infusion rate of 6 mL/h until full dilation of the cervix was achieved. If pain relief was not satisfactory 15 min after continuous infusion, parturients could receive a supplemental dose of 5 mL bolus via the epidural catheter. Subject pain was assessed with a 10-cm linear visual analogue scale (VAS), where 0 represented ‘no pain’ and 10 represented ‘most severe pain’. Pain scores were determined just before epidural placement and 5, 10, 15, 20, 25, 30, 60 min after epidural injection. Motor block was assessed by means of a modified four-grade Bromage scale (0 = able to lift extended leg at hip; 1 = able to flex knee but not lift extended leg; 2 = able to move foot only; and 3 = unable to move foot) (7).Each parturient received a 5 mL of 1% lidocaine as the testing and initial dose.

The following data were collected as demographic characteristics of the subjects: age at delivery, height, weight, and gestational age. Intraoperative hemodynamics including mean blood pressure (MAP) and heart rate (HR) at 5 min-intervals and pulse oxygen saturation were continuously monitored. Additional epidural analgesia agent requirements, instrument-assisted delivery, oxytocin use, and cesarean delivery were also recorded. Neonatal outcomes included fetal heart rate monitoring via a Doppler device, birth-weight, Apgar scores, and umbilical cord blood gas analysis. Immediately after delivery, with the placenta *in situ* and ideally prior to the neonate's first breath, an umbilical cord segment was isolated utilizing cord clamps. Umbilical arterial blood samples were collected using a 1 mL pre-heparinizedplastic syringe (Rapidlyte^™^; Bayer Corporation, EastWalpole, MA, USA) and a 21-gauge needle. Analysis was performed immediately after collection using a blood gas analyzer (GEM Premier 3000, Guangzhou, China). Maternal respiratory depression was defined as oxygen saturation below 95%. Other side-effects including nausea, vomiting, shivering, hypotension, vomiting, pruritus,and urinary retention were also recorded.

### Statistical analysis

Sample size calculation was performed according to a previously published study (8);29 parturients per group would have 80% power at the 5% significance level to detect a difference in umbilical arterial pH of 0.03 with standard deviation (SD) 0.04 for independent *t* test between the two groups. To allow for a possible drop-out rate of 10%–15%, a total of 66 parturients were enrolled in the present study. Values are expressed as number, mean and standard deviation (mean ± SD), or median (range). Student's unpaired *t* tests were used for continuous variables, and chi-square tests were used for categorical variables. Inter-group comparisons of maternal HR, MAP, and VAS scores and neonatal HR at the same time were tested using analysis of covariance. *p* < 0.05 was regarded as statistically significant. Statistical analysis was performed with the use of SPPS 16.0 for windows (SPSS Inc., Chicago, IL, USA).

## Results

### Demographic data

**Figure 1.**
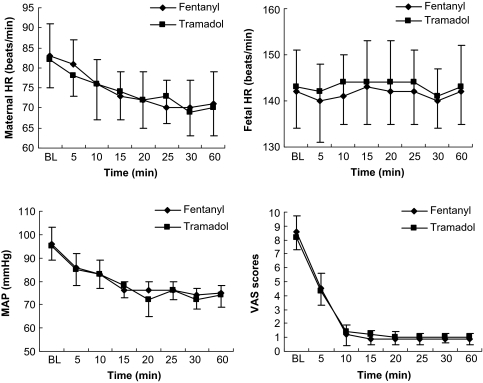
Changes in maternal HR, MAP, VAS scores, and fetal HR in each group. Values are expressed as mean ± SD. There was no significant difference between the two groups at any time points (*p* > 0.05).

Successful epidural block was achieved in 32 parturients in Group tramadol and 33 in Group fentanyl. Two parturients in Group tramadol and two parturients in Group fentanyl underwent cesarean delivery later. Therefore 30 parturients in Group tramadol and 31 parturients in Group fentanyl completed the present study. The two groups of parturients were compared with regard to maternal age, weight at term,height, gestational age, VAS scores, and stage of cervical dilation at base-line. Demographic variables and obstetric characteristics were similar between the two groups. Base-line VAS pain scores, cervical dilation, and fetal heart rate were also similar between the two groups (Table I and Figure 1).

**Table I.** Demographic data in each group.

	Group tramadol (*n* = 30)	Group fentanyl (*n* = 31)
Age (years)	26.0 ± 3.1	25.3 ± 2.6
Weight (kg)	69 ± 6	68 ± 7
Height (cm)	161 ± 5	160 ± 5
Gestational age (weeks)	39.2 ± 1.2	39.5 ± 1.3
Cervical dilatation (cm): start of study	3.5 ± 1.0	3.4 ± 0.9

Values are expressed as mean ± SD. There was no difference between the two groups.

### Maternal data

Maternal hemodynamic data are shown in Figure 1 and dermatomal sensory levels are presented in Table II. There was no significant difference in maternal MAP, HR, and VAS scores and neonatal HR between the two groups at any time points (*p* > 0.05). Furthermore, no parturients required additional epidural analgesia agent. Moreover, no significant difference was observed between the two groups in the percentages of subjects who were treated with oxytocin (*p* = 0.228). There was also no statistical significance in the rate of instrument-assisted delivery and rate of cesarean delivery between the two groups (*p* = 0.996 and *p* = 0.973, respectively).

**Table II.** Maternal data in each group.

	Group tramadol (*n* = 30)	Group fentanyl(*n* = 31)	*P* value
Nausea (*n*)	2	3	0.671
Vomiting (*n*)	0	1	0.508
Pruritus (*n*)	0	4	0.081
Shivering (*n*)	0	3	0.238
Urinary retention (*n*)	0	2	0.492
Respiratory depression (*n*)	0	0	–
Oxytocin use (*n*)	9	11	0.228
Instrument assisted delivery (*n*)	3	3	0.996
Cesarean delivery (*n*)	2	2	0.973
Additional requirements for epidural analgesic agent (*n*)	0	0	–
Hypotension (*n*)	0	0	–
Duration of first stage (min)	471 ± 143	451 ± 134	0.567
Duration of second stage (min)	50 ± 15	52 ± 16	0.480
Onset of sensory block (min)	4.4 ± 0.6	4.6 ± 0.6	0.181
Highest sensory block	T9 (T6–T10)	T8 (T6–T9)	0.353
Motor block:			
Maximum Bromage scale (*n*)0-1-2-3 (*n*)	24-6-0-0	23-8-0-0	0.590

Values are expressed as number, mean ± SD, or median (range). There was no difference between the two groups.

### Side-effects

Although no significant difference was seen, there was a tendency that the incidence of side-effects (pruritus, shivering, and urinary retention) was higher in Group fentanyl. No significant difference was observed in other side-effects such as nausea, vomiting, motor block, respiratory depression, and hypotension between the two groups (Table II). Furthermore, none of the parturients in any group required specific treatment during labor.

### Neonatal data

Neonatal data are presented in Table III. Birth-weight, umbilical arterial blood oxygen partial pressure and base deficit, lactate concentrations, and Apgar scores at 1 and 5 min were comparable between the two groups. Umbilical arterial blood pH was significantly lower, while arterial blood carbon dioxide partial pressure was higher in the fentanyl group than in the tramadol group (*p* = 0.001 and *p* = 0.026, respectively).

**Table III.** Neonatal data in each group.

	Group tramadol (*n* = 30)	Group fentanyl(*n* = 31)	*P* value
Birth-weight (kg)	3.4 ± 0.4	3.5 ± 0.4	0.465
Apgar scores			
1 min	9 (7–10)	9 (7–10)	0.608
5 min	10 (8–10)	10 (8–10)	0.751
Umbilical arterial blood acid–base status			
pH	7.27 ± 0.04	7.23 ± 0.04	0.001
PO_2_ (mmHg)	15.2 ± 4.2	15.0 ± 4.5	0.859
PCO_2_ (mmHg)	52 ± 6	57 ± 10	0.026
Base excess (mmol/L)	-3.0 ± 2.7	-3.9 ± 2.9	0.232
Lactate concentrations (mmol/L)	4.1 ± 1.1	4.3 ± 1.3	0.549

Values are expressed as mean ± SD or median (range). Umbilical arterial pH was significantly lower, while PaCO_2_ was higher in Group fentanyl than Group tramadol.

## Discussion

In this prospective, randomized, double-blinded study, we found that tramadol is as safe an adjunct as fentanyl to ropivacaine for pain management in labor epidural analgesia.

Pain management is essential for good obstetrical care, and effective analgesia is beneficial for both maternal and neonatal health (1). Epidural block is an attractive method for laboring analgesia. The epidural addition of opioid to local anesthetics has become a well accepted practice in the management of labor analgesia, since this modality can provide reasonable pain relief while decreasing the total dose of each agent, thus minimizing the relative side-effects. Fentanyl is a widely used opioid in clinical practice. However, the significant side-effects including pruritus, urinary retention, severe nausea and vomiting, and, occasionally, respiratory depression, are still unsettled (9). On the other hand, epidural administration of tramadol has not been well studied especially in labor analgesia. The analgesia effects of tramadol are mainly attributed to its dual mechanism of action as previously described (4). Added to this, its local anesthetic action may be another possible mechanism responsible for its analgesia effects (10). Importantly, it provided effective analgesia without significant respiratory, hemodynamic, or neurovirulent side-effects (5). The pharmacological profiles of tramadol along with minimal side-effects make it an attractive drug for epidural administration.

In the present study, comparative VAS scores obtained in both groups suggested that tramadol was as effective as fentanyl for labor epidural analgesia. Moreover, we could not find any difference in the onset time of sensory block between the two groups. However, prolongation of pain recognition after the epidural administration of tramadol in human study has been reported previously (11). Our own preliminary studies also demonstrated that the onset time of anesthesia of tramadol was longer than fentanyl. Here, we used lidocaine as the testing dose (1% and 5 mL solution), a routine dosage regime at our institution, to make up for the slow onset time of sensory block of tramadol. Furthermore, the intensity of motor block, as reflected by Bromage motor scale scores, was similar between the two groups. These results were also supported by previous studies (12), in which they showed that ropivacaine 0.125% produced a low incidence of motor block.

With regard to the side-effects, there was a tendency that the incidence of shivering was higher in parturients who received fentanyl, a mechanism that might be mediated by its serotonergic or noradrenergic activity (13). Shivering causes distress and increased oxygen consumption to the parturients and thus may induce arterial hypoxemia. Moreover, pruritus and urinary retention seemed to be higher in Group fentanyl, although no significant difference was seen between the two groups. As the present study is a pilot study, more studies with larger sample sizes are suggested to test these observations.

Epidural use of fentanyl may reintroduce the problem of neonatal depression just as when it was used intravenously (14). Though the Apgar scoring system is a valuable surrogate for assessment of neonatal well-being immediately after birth (15), a growing body of evidence suggests that umbilical arterial pH may provide objective documentation to assess intra-partum care (16). More recently, White et al. (17) even showed that introduction of universal umbilical cord blood gas analysis may result in improved perinatal outcomes.The results of the present study demonstrated that Apgar scores at 1 and 5 min in all the neonates were more than 7. However, we observed that fentanyl use was associated with a higher PaCO_2_ and a lower umbilical cord blood pH values than tramadol use.

There are three obvious limitations in the present study. First, pH is an objective retrospective measure of the fetal exposure and response to hypoxia during labor. The timing of cord blood clamping therefore seems to be critical for the interpretation of cord blood gases that may be involved in the processes and procedures. But this bias was unlikely because the procedure was performed by the same midwife. Second, the analgesic potency ratio between epidural administration of tramadol and fentanyl remains unclear. Previous study has suggested that the analgesic potency ratio of fentanyl and tramadol is nearly 1:1000 after intramuscular injection (18). Our own preliminary study showed that fentanyl 5 μg/mL is associated with more side-effects and tramadol 5 mg/mL had similar analgesic efficacy to fentanyl 3 μg/mL. Therefore, we chose the final doses of tramadol 5 mg/mL and fentanyl 3 μg/mL in the present study. Third, although epidural administration of tramadol has been extensively used for analgesia by numerous investigators in clinical studies, more studies are needed to assess the safety for its neurotoxicity.

In conclusion, we found that tramadol 5 mg/mL had similar analgesia efficacy to fentanyl 3 μg/mL with fewer maternal and neonatal side-effects, suggesting that tramadol may be a safe alternative to fentanyl as an adjunct for labor analgesia.
